# Trends in Israeli community-based opioid prescribing, 2010–2020, an observational study of the country’s largest HMO

**DOI:** 10.1186/s13584-023-00581-w

**Published:** 2023-11-16

**Authors:** Reuven L. Dressler, Ehud Kaliner, Matan J. Cohen

**Affiliations:** 1 Clalit Health Services, Department of Family Medicine, Hebrew University of Jerusalem Faculty of Medicine, Jerusalem District, HaGitit 64-B, 9839037 Maale Adumim, Israel; 2 State of Israel Ministry of Health, Central District, 91 Herzl St., 7243003 Ramla, Israel; 3 Clalit Health Services, Hebrew University of Jerusalem Faculty of Medicine, Jerusalem District, 1 Ygal Alon St., Bet Shemesh, Israel

**Keywords:** Opioid use, Opioid misuse, Opioid addiction, Prescription medicine addiction

## Abstract

**Background:**

Prescription opioids are widely used for pain control and palliative care but have been associated with a variety of untoward effects, including opioid use disorder, addiction, and increased mortality. Patterns of opioid use in Israel are to date poorly described.

**Methods:**

Using a community-based database, the authors performed a retrospective analysis of filled opioid prescriptions of Israeli HMO members 18 years of age or older during the years of 2010–2020 that filled at least one opioid prescription. Morphine milligram equivalent (MME) calculations were stratified by presence or absence of oncology diagnosis and by specific opioid medication.

**Results:**

The percentage of HMO members who filled at least one opioid prescription increased every year from 2.1% in 2010 to 4.2% in 2020. There was an increase in the MME per prescription (44.2%), daily MME per capita (142.1%) and MME per prescription-filling patient (39%) from 2010 to 2020. Increased prescription opioid use is driven by a small group of non-oncological patients, which is less than 1.5% of opioid-prescribed patients and 0.1% of the adult population, primarily owing to fentanyl use.

**Conclusion:**

Supervision and control of opioid prescriptions in Israel should be a focused effort directed at patients prescribed uniquely high dosages rather than a population-wide strategy that focuses on all patients prescribed opioids. This should be complemented by improved physician training and access to non-opioid therapies, as well as improved data collection and analysis.

**Supplementary Information:**

The online version contains supplementary material available at 10.1186/s13584-023-00581-w.

## Introduction

Prescription opioids are widely prescribed by medical practitioners for pain control and palliative care [1–3]. Worrisome trends of increasing use have been documented over the past decades in high-income countries, contributing to both morbidity and mortality [4–6]. Alongside their primary goal of alleviating pain, prescription opioids have been proven to be associated with a variety of untoward effects, including opioid use disorder, addiction, and increased early mortality [6–9].

Less is known about the patterns of prescription opioid use in Israel than in other developed nations. However, since the beginning of the twenty-first century, an increase in opioid prescription has been observed on the national-level [10], and in Israel’s four Healthcare Medical Organizations (HMOs) [11–15]. To date there are no Israeli guidelines in place dedicated to the use of opioids, nor are there regulations set to supervise or control opioid use.

The authors examined opioid prescribing patterns in one of Israel’s HMOs over a 10-year span and sought to define whether the overall increased opioid prescription is widespread and uniform or limited to a select group which drives the national pattern. This data should inform both national and HMO health policy efforts to restrain opioid prescription.

## Methods

The data presented in this report derives from the Clalit Health Services (CHS) data repository. CHS in the largest healthcare provider in Israel, serving over half of the population, totalling just under five million individuals. All CHS members above 18 years of age who had at least one opioid prescription filled between 2010 and 2020 were included in the analysis after obtaining authorization from the CHS ethics’ institutional review board (COM1-159-19). The dataset used for this study was anonymized and there was no requirement for participant consent. Opioids were classified according to Anatomical Therapeutic Chemical Classification System (ATC) level 5 opioid ingredient (buprenorphine, fentanyl, morphine, oxycodone and tramadol). All formulations including these ingredients. Codeine was excluded as its use was both common and stable with no effect on larger trends. Excluded were also propoxyphene and pethidine that were prescribed infrequently. There are no other opioids registered for use and prescription in the medical healthcare system of Israel.

Patients were classified as oncology patients if they were ever diagnosed with solid malignancy, leukemia or lymphoma if at any period during their lifetime they were diagnosed with these hemato-oncological diseases. This diagnosis is linked to the National Cancer Registry [16], thus, if they were diagnosed when under the care of a different HMO (other than CHS) this diagnosis would still be registered on their patient record. Having been diagnosed with a hemato-oncological disease entitles patients to various social benefits and allowances, thus such diagnoses are cross referenced between the National Cancer Registry, the National Insurance Agency and the HMOs, with verification of clinic-pathological findings.

Morphine milligram equivalent (MME) for each filled prescription was calculated according to accepted methods [17]. Calculation of MME allows comparisons of opioid medication and standardization of potency. For example, 150 mg of tramadol are equivalent to 30 MME, as are 30 mg of morphine, or 20 mg of oxycodone. Daily MME was calculated by summing the total MME of filled prescriptions and dividing this total by the length of follow-up from first filling until censorship or death. Patients were stratified to three unique patient groups; oncology patients, non-oncology patients who filled 90 or more daily MME, and non-oncology patients who filled less than 90 daily MME.

We present the findings over time with percentages and ratios of total dispensed MMEs: MME per prescription-filling patient and MME per capita were calculated as two measures of total MME dispensed. The latter is presented in order to control for the natural growth of CHS member population over time (approximately 1.6% per year).

## Results

Opioid prescriptions were filled by 1,063,820 CHS members between January 2010 and December 2020, of which 194,815 (18.3%) were classified as oncology patients. The percentage of CHS members who filled at least one opioid prescription increased every year from 2.1% in 2010, to 4.4% in 2017, plateaued in 2018 and 2019, and decreased to 4.2% in 2020. The analysis included 7,850,549 filled opioid prescriptions, totaling 5,420,088,568 MME. There was an increase in the MME per prescription (44.2%), daily MME per capita (142.1%) and MME per prescription-filling patient (39.1%) (Table 1).

**Table 1:** Total numbers of opioid prescriptions, patients filling opioid prescriptions, and dispensed morphine milligram equivalents (MME)

Year	Total opioid prescriptions	Patients who filled opioid prescriptions	Total MME dispensed	MME per prescription*	Daily MME per prescribed patient**	Daily MME per capita***
2010	442,602	117,626	273,045,916	617	6.4	0.19
2011	485,556	130,054	307,900,039	634	6.5	0.21
2012	537,570	150,559	338,264,350	629	6.2	0.23
2013	616,684	180,652	372,171,397	604	5.6	0.24
2014	694,575	207,984	403,087,485	580	5.3	0.26
2015	754,508	224,595	448,705,483	595	5.5	0.28
2016	802,379	235,313	507,146,315	632	5.9	0.32
2017	858,186	247,700	591,209,343	689	6.5	0.36
2018	880,096	247,619	671,528,153	763	7.4	0.41
2019	898,256	251,092	724,027,460	806	7.9	0.43
2020	880,137	240,218	783,002,625	890	8.9	0.46

*Total MME/total number of prescriptions

**Total MME/(365*Patients who received opioids)

***Total MME/(365*number of insured patients in Clalit Health Services)

**Fig. 1:**
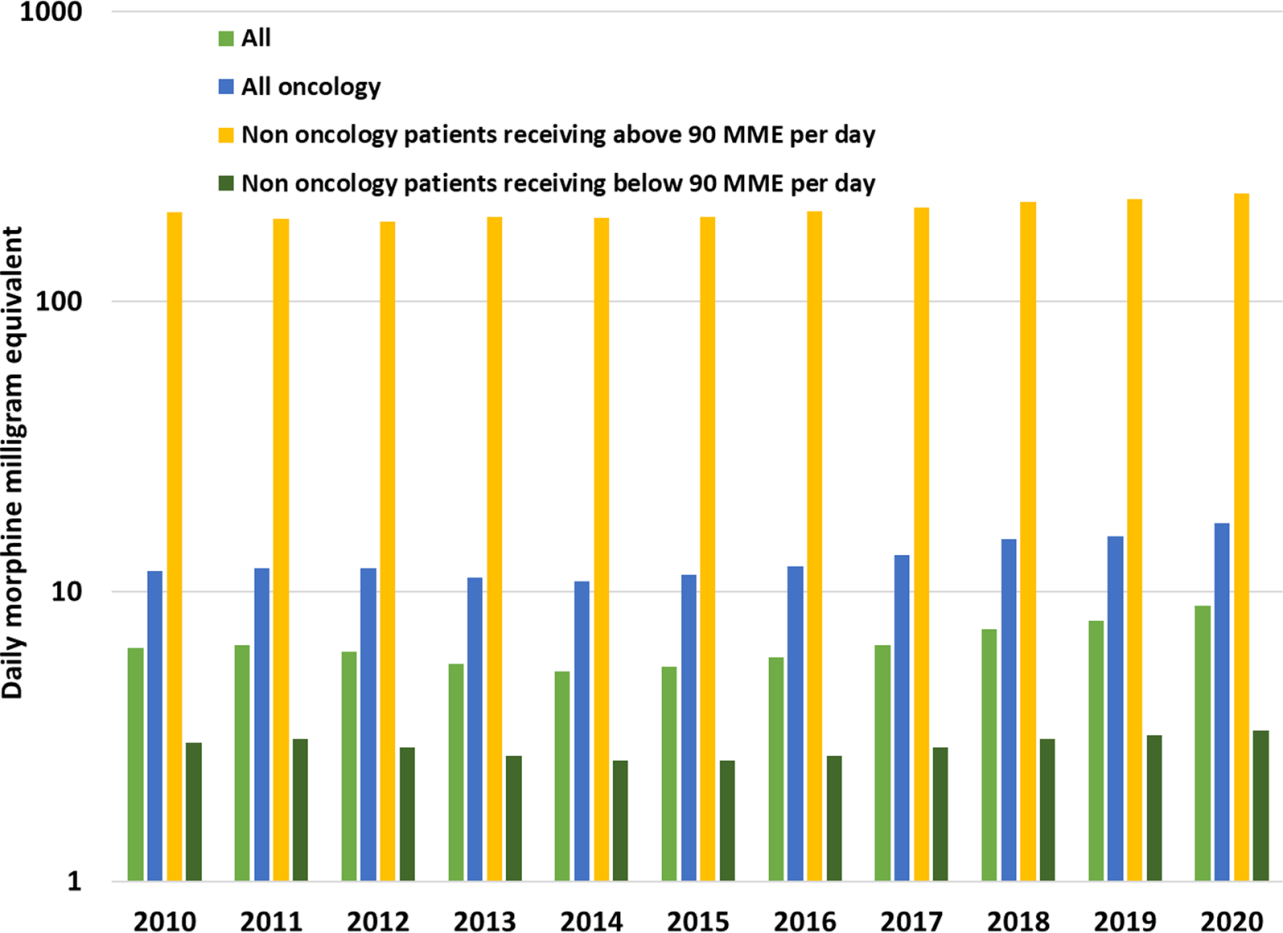
Daily MME filled by all patients, all oncology patients, non-oncology patients receiving 90 or more daily MME daily, or less than 90 daily MME

Average daily MME per prescription-filling patient increased among oncology patients from 11.7 to 17.2 daily MME (46.4%) between 2010 and 2020. This measure among non-oncology patients receiving less than 90 daily MME (n = 84,578 in 2010 and n = 195,773 in 2020) increased from 3.0 to 3.4 daily MME (10.0%) and among non-oncology patients filling 90 or more daily MME (n = 540 in 2010 and n = 3318 in 2020) from 202.7 to 235.7 daily MME (16.0%), far greater than that received by oncology patients (n = 32,508 in 2010 and n = 41,127 in 2020), in absolute terms (Fig. 1).

**Fig. 2:**
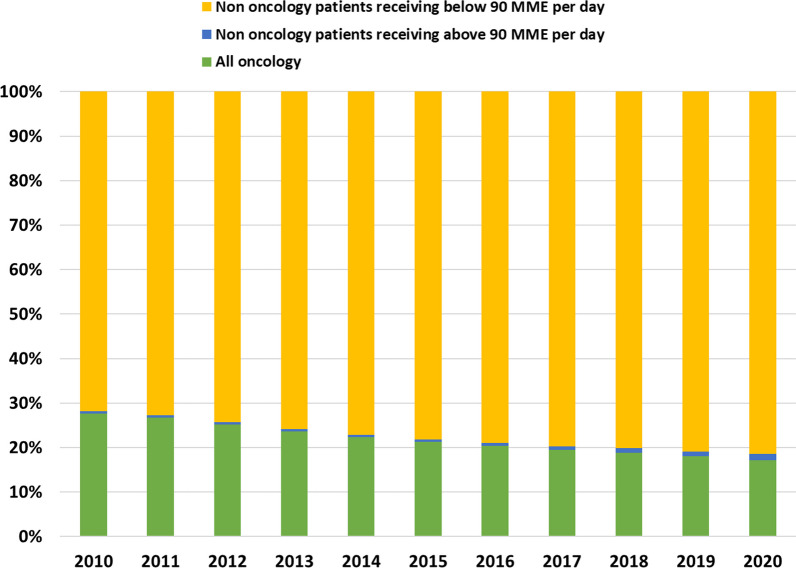
Percentages of total population that filled an opioid prescription by all oncology patients, non-oncology patients receiving 90 or more MME or below 90 daily MME (excluding codeine, pethidine and propoxyphene)

The marked contribution of the non-oncology patients receiving 90 or more daily MME contrasts the group’s small absolute size (Fig. 2). In 2010 they constituted 0.46% of all opioid filling patients, and by 2020 they were 1.3%. Oncology patients filling opioid prescriptions decreased from 27.6% of all patients in 2010 to 17.1% in 2020.

**Fig. 3:**
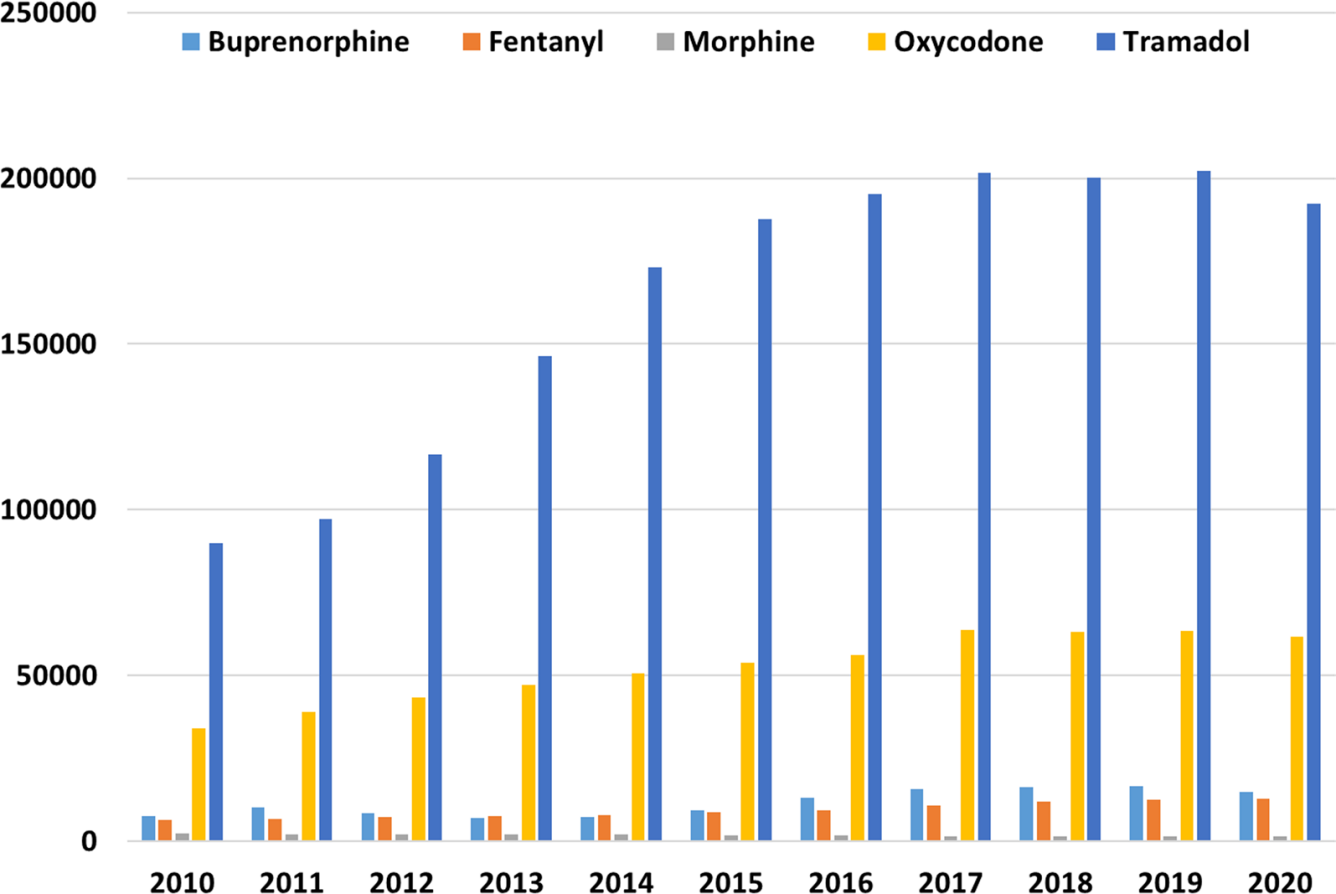
Number of patients who filled at least one opioid prescription by year

During the study period, 953,726 patients filled at least one prescription of tramadol, 333,107 oxycodone, 82,893 buprenorphine, 63,746 fentanyl and 14,977 morphine. These increases were consistent for most opioids studied, across each year between 2010 and 2020. The largest increase in the number of patients prescribed an opioid was for tramadol (113%), followed by fentanyl (103%), buprenorphine (91%), and lastly oxycodone (81%); whereas morphine decreased 31% (Fig. 3).

**Fig. 4:**
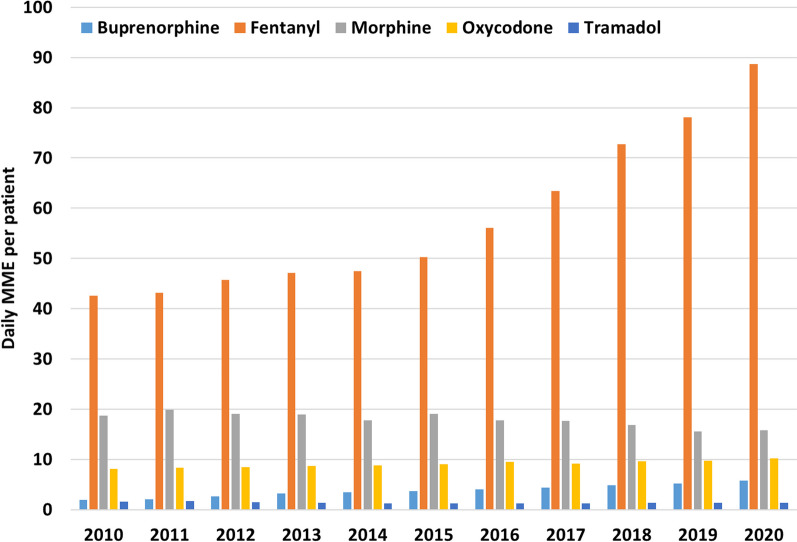
Average daily MME filled by each patient that received at least one opioid prescription per medication type and per year

Most of the daily MME per opioid prescribed patient was comprised of fentanyl MMEs, followed by morphine, oxycodone, buprenorphine, and lastly tramadol. Fentanyl also had the sharpest increase over the study period—46.1 daily MME per patient (108%), followed by buprenorphine—3.8 daily MME per patient (194%) and oxycodone—2.1 daily MME per patient (26%). Decreases were seen with morphine (− 2.86 daily MME per patient, 15% decrease) and tramadol (− 0.26 daily MME per patient, 15% decrease) (Fig. 4).

**Fig. 5:**
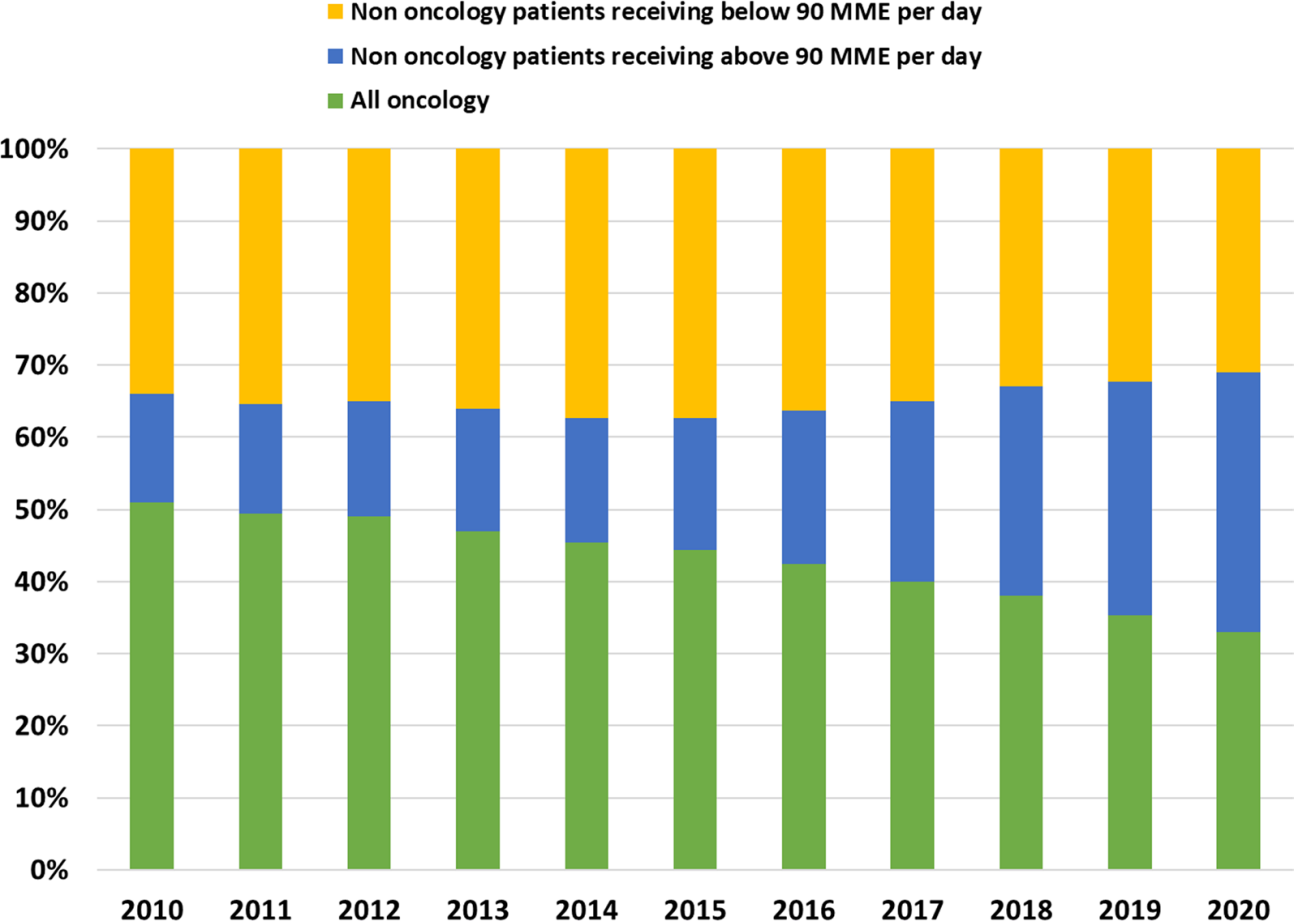
Percentage of total annual MME of filled prescriptions from patients that filled at least one opioid prescription by all oncology patients, non-oncology patients receiving 90 or more daily MME, or less than 90 daily MME

**Fig. 6:**
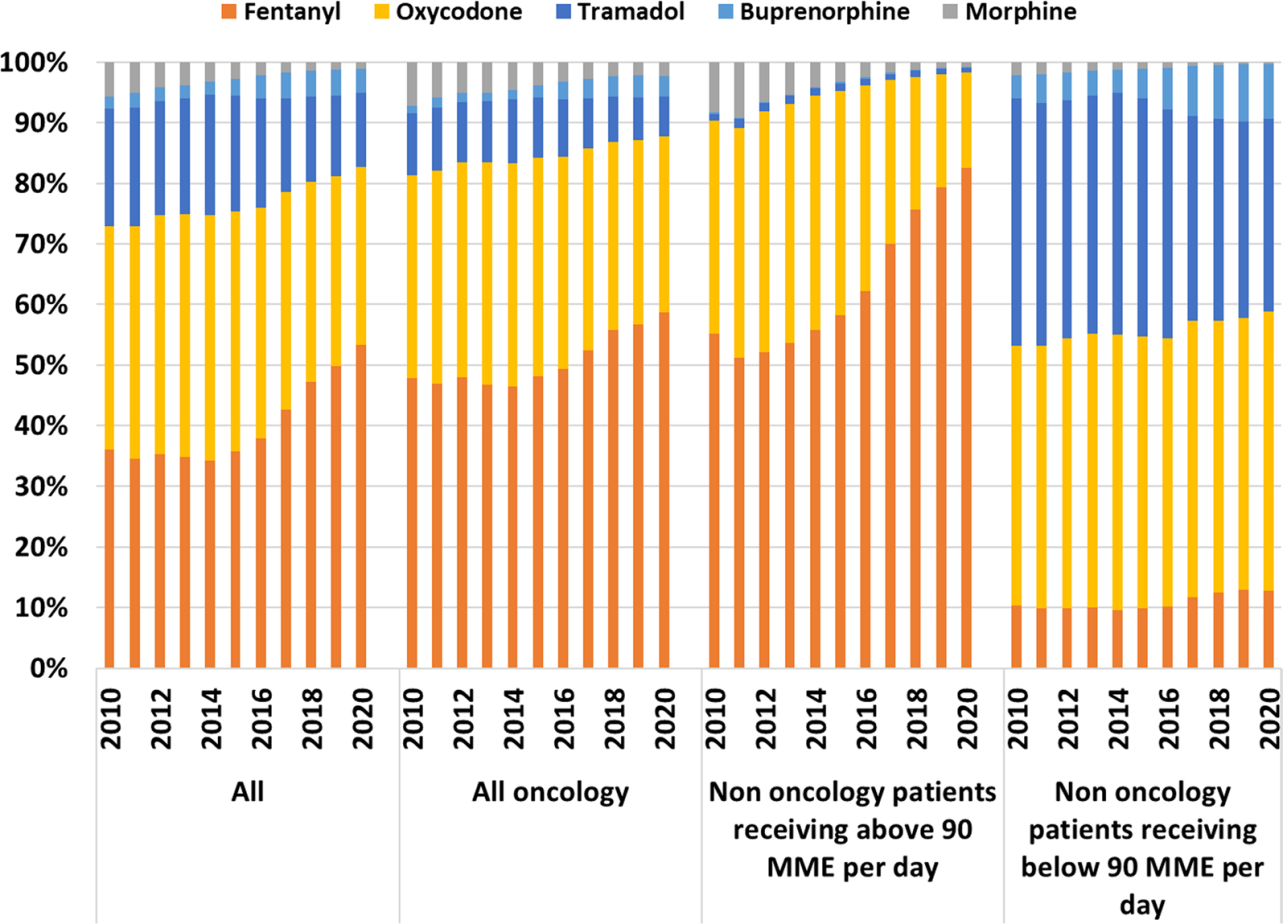
Distribution of total MME dispensed per year, by all patients, all oncology patients, non-oncology patients receiving 90 or more daily MME or less than 90 daily MME

The percentage of total annual MME of filled prescriptions according to the three patient groups is presented in Fig. 5. In 2010 oncology patients filled 51% of total MME and in 2020 they filled 33% of total MME. The respected percentages for non-oncology patients filling 90 or more daily MME were 15% in 2010 and 36% in 2020. Finally, the percentage of MME in each patient group, per opioid medication demonstrates the increasing filling of fentanyl prescriptions among non-oncology patients filling 90 or more daily MME (Fig. 6 and Additional file 1: Table S1). Fentanyl MME among non-oncology patients filling 90 or more daily MME was the most pronounced prescription, driving the overall trend of the increased use of opioids.

## Discussion

This report shows clear and consistent increases of the number of total opioid prescriptions, number of patients receiving at least one opioid prescription per annum, and average MME filled per patient. Daily MME per capita, which controls for CHS membership growth, increased as well. These findings echo other reports worldwide [18, 19] and also from Israel [11–15].

Between 2010 and 2020 the number of non-oncology patients filling 90 or more daily MME increased (over sixfold) from 540 to 3318 patients (0.4–1.3% of opioid filling patients). This small group’s quantity of filled MMEs surpassed that of all oncology patients by 2020. By this year, 36% of the total dispensed MME were prescribed to this small fraction of patients. Fentanyl prescriptions are fueling the growth of filled MME for all patients receiving an opioid, and this is dominated by the large and increasing use of fentanyl among non-oncology patients receiving 90 or more MME. In 2020, 82% of the MME dispensed to these patients were fentanyl. Thus, in 2020, when the CHS population amounted to 4.6 million individuals, of whom 240 thousand individuals received at least one opioid prescription, 29% of the total amount of MME dispensed were fentanyl prescribed to just over 3000 patients.

This analysis is limited to opioids that were filled by CHS or affiliated private pharmacies. Missing are filled prescriptions from non-affiliated private pharmacies, hospital-based acute care or inpatient settings, and addiction treatment centers. These missing prescriptions have little impact on the patterns presented due to the widespread availability of primary care health services, to whom all residents are entitled with relatively little associated costs. Missing prescriptions would under-estimate the true amount of filled opioid prescriptions, but would have to differ between opioid types to influence the trends demonstrated. For example, if missing prescription were far more common among oncology patients and non-oncology patient receiving less than 90 MME per day, this report’s assessment of the percentage of MME prescribed to non-oncology patients receiving more than 90 MME per day would be overestimated. If some specific medication types were missed more often than others, the percentages of these medication of the total MME dispensation would be underestimated.

This Israeli community-based analysis shows trends of increasing prescriptions through the second decade of the twenty-first century, that significantly differ from those of the USA, where decreases in opioid prescribing are seen over the same time period [20, 21]. We believe that there are a number of contributing factors to these trends, of increasing prescriptions, that are acting synergistically. As has been reported in the Israeli Parliament, currulica from Israeli medical schools and relevant accredited residency programs do not contain topics such as addiction or prescription drug misuse and abuse [22]. Israel also allows for any physician with an active medical license to prescribe any opioid, in any dose, as the physician may see fit. This combination of inappropriate knowledge and skill, coupled with no administrative oversight contributed to the opioid epidemic burdening healthcare systems. Furthermore, data collection so far, including this report and others [13, 23, 24] has been retrospective and conclusions and insights, counterfactual. Without accurate real-time data collection and reliable real-time monitoring of opioid prescribing, health ministry and HMO administrators are unable to curtail opioid prescribing. Implementation of such surveillance tools in CHS and indeed in all care-providers should be relatively simple to introduce, as most prescriptions today are digitized and few are handwritten. Yet policy regarding the methods of institutional surveillance needs to consider their focus: physicians prescribing a lot or large doses, patients being prescribed large dosages, high-dose prescriptions, specific compounds, etc. It either case, the technological abilities exist; their implementation needs only conviction and leadership to advance.

This report and the presented data, though robust, have several limitations worth considering. The first, as mentioned above, is the reliance on registered prescriptions alone, not considering private pharmacy dispensations and private “white” prescriptions. Second, this report covers half of the insured Israelis within a single large healthcare provider. It would be beneficial to examine prescriptions in other providers and those provided from hospitals in order to identify similar or other patterns and trends in opioid compound choice and dosages dispensed throughout the decade. Third, this report identifies a relatively small group of patients as “drivers” of the epidemic. A study of prescription patterns by physicians would complement these results, perhaps identifying frequent high dose prescribers among whom focused interventions could be directed. A fourth limitation results from the absence of patient specific data regarding co-morbidities and prescription indications, which might identify patients who could benefit from, and be referred to, alternative pain relief interventions, thereby reducing prescription by improving care strategies, rather and imposing limitations of prescriptions without offering substitute modalities. This information would be most valuable in order to better understand the group we direct focus to in this report, non-oncology patients receiving high doses of opiates and specifically fentanyl.

While difficult to quantify, the medical culture of Israel is relatively consumer-based. Israeli citizens who are residents receive membership in one of the country’s four HMOs. Primary care is offered by community-based clinics, with vibrant competition between HMOs for members. In our (the authors’) experience and review of medical records during our clinical work we observed that physicians often prescribe opioid analgesia without first discussing the relative risks and benefits of both short- and long-term opioid therapy and, on occasion, prescribe opioids simply at request of their patients.

Though both patients and healthcare providers in Israel are to an extent aware of the challenges of opioid use in the USA and other Western counties, Israel has not assessed or reported, to date, associations of opioid use and increased morbidity and mortality from opioid poisonings, overdose or death as seen in the USA [25, 26]. In fact, this may be due to inaccurate data documentation in the state and medical records. Autopsies in some populations are not permitted by family members and not common practice in Israel, limiting post-mortem evaluation. Additionally, standard urine toxicology testing in hospital settings as well as post-mortem medical examination do not include testing for fentanyl and oxycodone, only methadone and morphine [27]. Owing to cultural taboos, physicians may be reluctant to document opioids as a contributing cause of death. Physicians might be fearful of stigma (real or perceived) which might impact memories, closure and acceptance of the bereaved family and community to which the deceased is associated with.

## Conclusions

This report identifies that non-oncology patients receiving high-dose opioids are the driver of increased prescription opioid use over the past decade. Prescription fentanyl use is increasing and accounts for over 80% of the filled MME in non-oncology patients. The overall trend of increased opioid use is driven by about 3,000 patients out of 4.6 million CHS members. Health policy intended to curb overuse and misuse should be focused on this select group, offering non-opioid therapies (i.e., physical therapy, cognitive-behavioral therapy, injections, non-opioid medications) to prevent opioid dose escalation that may not otherwise be indicated. Pain medicine and addiction prevention should be incorporated into medical school and residency curricula as well as become a requirement for maintenance of medical licensure. Both the Israeli Health Ministry and national health maintenance organizations must increase surveillance, reporting, and perhaps curtail prescription of opioids. Further research is required to describe in greater detail the phenomenon of prescription opioid use in Israel including the examination of geographical variation, clinical indications used or misused, patient and physician behaviors and, financial and clinical consequences and incentives. Policy changes must balance the call to ease patient pain and suffering with the unwanted consequences of opioid overuse and counterproductive implications to patients and society.

### Supplementary Information

**Additional file 1:** Supplementary Table.

## Data Availability

Data not available.

## Abbreviations

MME: Morphine milligram equivalent
HMO: Health Medical Organizations
CHS: Clalit Health Services
