# Supplementary material for: Trends in Israeli community-based opioid prescribing, 2010–2020, an observational study of the country’s largest HMO
Source: Isr J Health Policy Res. 2023 Nov 16;12:34. doi: 10.1186/s13584-023-00581-w (PMC10652579; doi:10.1186/s13584-023-00581-w)
Supplement: Supplementary file 1 — Additional file 1: Supplementary Table. [file 13584_2023_581_MOESM1_ESM.pdf]

Supplementary table

Supplementary table 1. Total MME dispensed per year, by all patients, all oncology patients, non-oncology patients receiving 90 or more daily MME or less than 90 daily MME.

		Total MME filled						Percents of MME per medication group				
		Buprenorphine	Fentanyl	Morphine	Oxycodone	Tramadol	Total	Buprenorphine	Fentanyl	Morphine	Oxycodone	Tramadol
All	2010	5,501,766	98,549,045	15,271,635	100,730,880	52,992,590	273,045,916	2.0%	36.1%	5.6%	36.9%	19.4%
	2011	7,682,912	106,157,170	15,327,655	118,578,203	60,154,100	307,900,040	2.5%	34.5%	5.0%	38.5%	19.5%
	2012	8,080,250	119,519,215	13,864,975	133,148,078	63,651,833	338,264,350	2.4%	35.3%	4.1%	39.4%	18.8%
	2013	8,042,790	129,378,345	14,123,310	149,659,598	70,967,355	372,171,398	2.2%	34.8%	3.8%	40.2%	19.1%
	2014	8,909,740	137,937,795	12,931,945	163,175,183	80,132,823	403,087,485	2.2%	34.2%	3.2%	40.5%	19.9%
	2015	12,367,390	160,558,801	12,020,475	177,296,430	86,462,388	448,705,484	2.8%	35.8%	2.7%	39.5%	19.3%
	2016	19,319,580	191,978,426	11,239,105	193,325,948	91,283,258	507,146,316	3.8%	37.9%	2.2%	38.1%	18.0%
	2017	25,290,020	251,580,241	10,034,545	213,433,200	90,871,338	591,209,344	4.3%	42.6%	1.7%	36.1%	15.4%
	2018	28,912,520	316,803,407	9,298,045	221,742,593	94,771,589	671,528,153	4.3%	47.2%	1.4%	33.0%	14.1%
	2019	31,706,710	360,671,975	8,644,245	226,543,583	96,460,948	724,027,460	4.4%	49.8%	1.2%	31.3%	13.3%
	2020	30,887,570	417,752,405	8,829,475	230,449,350	95,083,825	783,002,625	3.9%	53.4%	1.1%	29.4%	12.1%
		Total MME filled						Percents of MME per medication group				
		Buprenorphine	Fentanyl	Morphine	Oxycodone	Tramadol	Total	Buprenorphine	Fentanyl	Morphine	Oxycodone	Tramadol
All oncology	2010	1,767,160	66,769,165	9,947,940	46,658,895	14,283,560	139,426,720	1.3%	47.9%	7.1%	33.5%	10.2%
	2011	2,408,946	71,411,330	8,905,010	53,617,748	15,879,385	152,222,419	1.6%	46.9%	5.8%	35.2%	10.4%
	2012	2,615,020	79,544,805	8,256,095	58,920,443	16,434,013	165,770,375	1.6%	48.0%	5.0%	35.5%	9.9%
	2013	2,569,350	81,545,520	8,760,425	64,091,348	17,544,264	174,510,906	1.5%	46.7%	5.0%	36.7%	10.1%
	2014	2,955,400	84,454,174	8,269,145	67,293,705	19,162,875	182,135,299	1.6%	46.4%	4.5%	36.9%	10.5%
	2015	4,138,470	95,950,618	7,572,880	72,001,778	19,884,764	199,548,509	2.1%	48.1%	3.8%	36.1%	10.0%
	2016	6,270,600	105,434,776	6,850,815	74,568,278	20,299,279	213,423,747	2.9%	49.4%	3.2%	34.9%	9.5%
	2017	7,621,880	123,025,704	6,323,220	78,340,050	19,301,690	234,612,544	3.2%	52.4%	2.7%	33.4%	8.2%
	2018	8,723,540	143,335,422	5,983,900	79,720,980	19,009,633	256,773,474	3.4%	55.8%	2.3%	31.0%	7.4%
	2019	9,154,740	145,201,436	5,569,750	77,970,000	18,374,370	256,270,296	3.6%	56.7%	2.2%	30.4%	7.2%

	2020	8,549,380	151,517,650	5,951,760	75,073,425	17,232,675	258,324,890	3.3%	58.7%	2.3%	29.1%	6.7%
		Continued										
		Total MME filled						Percents of MME per medication group				
		Buprenorphine	Fentanyl	Morphine	Oxycodone	Tramadol	Total	Buprenorphine	Fentanyl	Morphine	Oxycodone	Tramadol
Non oncology patients receiving above 90 MME per day	2010	80,830	22,041,060	3,329,250	14,068,493	440,730	39,960,363	0.2%	55.2%	8.3%	35.2%	1.1%
	2011	106,720	24,073,360	4,292,835	17,836,058	676,410	46,985,383	0.2%	51.2%	9.1%	38.0%	1.4%
	2012	52,980	28,214,300	3,570,450	21,540,345	724,285	54,102,360	0.1%	52.1%	6.6%	39.8%	1.3%
	2013	76,580	34,511,737	3,489,055	25,382,040	843,968	64,303,380	0.1%	53.7%	5.4%	39.5%	1.3%
	2014	106,680	39,164,898	2,853,940	27,195,720	880,103	70,201,341	0.2%	55.8%	4.1%	38.7%	1.3%
	2015	156,940	48,289,310	2,709,985	30,648,465	1,042,350	82,847,050	0.2%	58.3%	3.3%	37.0%	1.3%
	2016	305,060	67,753,907	2,684,475	37,019,378	1,254,110	109,016,930	0.3%	62.1%	2.5%	34.0%	1.2%
	2017	536,900	104,404,786	2,445,475	40,538,265	1,320,186	149,245,612	0.4%	70.0%	1.6%	27.2%	0.9%
	2018	461,580	145,810,309	2,306,700	42,264,720	1,844,140	192,687,449	0.2%	75.7%	1.2%	21.9%	1.0%
	2019	479,640	185,209,014	2,180,790	43,480,973	1,964,284	233,314,700	0.2%	79.4%	0.9%	18.6%	0.8%
	2020	776,650	235,485,727	1,975,135	45,256,103	1,953,406	285,447,021	0.3%	82.5%	0.7%	15.9%	0.7%
		Total MME filled						Percents of MME per medication group				
		Buprenorphine	Fentanyl	Morphine	Oxycodone	Tramadol	Total	Buprenorphine	Fentanyl	Morphine	Oxycodone	Tramadol
Non oncology patients receiving below 90 MME per day	2010	3,653,776	9,738,820	1,994,445	40,003,493	38,268,300	93,658,834	3.9%	10.4%	2.1%	42.7%	40.9%
	2011	5,167,246	10,672,480	2,129,810	47,124,398	43,598,305	108,692,239	4.8%	9.8%	2.0%	43.4%	40.1%
	2012	5,412,250	11,760,110	2,038,430	52,687,290	46,493,535	118,391,615	4.6%	9.9%	1.7%	44.5%	39.3%
	2013	5,396,860	13,321,088	1,873,830	60,186,210	52,579,124	133,357,112	4.0%	10.0%	1.4%	45.1%	39.4%
	2014	5,847,660	14,318,723	1,808,860	68,685,758	60,089,845	150,750,846	3.9%	9.5%	1.2%	45.6%	39.9%
	2015	8,071,980	16,318,873	1,737,610	74,646,188	65,535,274	166,309,924	4.9%	9.8%	1.0%	44.9%	39.4%
	2016	12,743,920	18,789,743	1,703,815	81,738,293	69,729,869	184,705,639	6.9%	10.2%	0.9%	44.3%	37.8%
	2017	17,131,240	24,149,752	1,265,850	94,554,885	70,249,461	207,351,188	8.3%	11.6%	0.6%	45.6%	33.9%
	2018	19,727,400	27,657,676	1,007,445	99,756,893	73,917,816	222,067,230	8.9%	12.5%	0.5%	44.9%	33.3%
	2019	22,072,330	30,261,525	893,705	105,092,610	76,122,294	234,442,464	9.4%	12.9%	0.4%	44.8%	32.5%
	2020	21,561,540	30,749,028	902,580	110,119,823	75,897,744	239,230,714	9.0%	12.9%	0.4%	46.0%	31.7%
